# Combined Bone Marrow and Kidney Transplantation for the Induction of Specific Tolerance

**DOI:** 10.1155/2016/6471901

**Published:** 2016-04-30

**Authors:** Yi-Bin Chen, Tatsuo Kawai, Thomas R. Spitzer

**Affiliations:** ^1^Bone Marrow Transplant Unit, Department of Medicine, Massachusetts General Hospital, Boston, MA 02114, USA; ^2^Transplantation Unit, Department of Surgery, Massachusetts General Hospital, Boston, MA 02114, USA

## Abstract

The induction of specific tolerance, in order to avoid the detrimental effects of lifelong systemic immunosuppressive therapy after organ transplantation, has been considered the “Holy Grail” of transplantation. Experimentally, tolerance has been achieved through clonal deletion, through costimulatory blockade, through the induction or infusion of regulatory T-cells, and through the establishment of hematopoietic chimerism following donor bone marrow transplantation. The focus of this review is how tolerance has been achieved following combined bone marrow and kidney transplantation. Preclinical models of combined bone marrow and kidney transplantation have shown that tolerance can be achieved through either transient or sustained hematopoietic chimerism. Combined transplants for patients with multiple myeloma have shown that organ tolerance and prolonged disease remissions can be accomplished with such an approach. Similarly, multiple clinical strategies for achieving tolerance in patients without an underlying malignancy have been described, in the context of either transient or durable mixed chimerism or sustained full donor hematopoiesis. To expand the chimerism approach to deceased donor transplants, a delayed tolerance approach, which will involve organ transplantation with conventional immunosuppression followed months later by bone marrow transplantation, has been successful in a primate model. As combined bone marrow and organ transplantation become safer and increasingly successful, the achievement of specific tolerance may become more widely applicable.

## 1. Introduction

The “Holy Grail” of solid organ transplantation is the induction of donor specific immunological tolerance in order to avoid the complications of long-term systemic immunosuppressive therapy. Tolerance is defined by the absence of a destructive immune response following transplantation in the absence of systemic immunosuppression. Specific tolerance refers to acceptance of the allograft with preservation of third-party immunity. The induction of tolerance has been attempted experimentally in a number of ways. These ways include clonal deletion [1], costimulatory blockade [2], the induction or infusion of regulatory T-cells [3, 4], and the establishment of mixed chimerism following donor bone marrow transplantation [5–10].

Several pathways to clinical tolerance have also been demonstrated. A minority of patients who have stopped their systemic immunosuppressive therapy (usually on their own) have subsequently had normal allograft function. This so-called spontaneous tolerance has been reported in up to 20% of liver transplant recipients, but only rarely in recipients of a kidney transplant [11, 12]. There are also numerous anecdotes of patients who received a kidney transplant after a prior hematopoietic cell transplant for a hematologic malignancy or other life-threatening blood disorder from the same donor [13]. These recipients have expectedly accepted the kidney allograft without systemic immunosuppression. More recently combined organ and bone marrow transplantation with the achievement of mixed or full donor chimerism has been shown to be a method of achieving specific tolerance (Table 1) [14–19].

The focus of this review will be the induction of specific tolerance through hematopoietic chimerism following combined bone marrow and kidney transplantation.

## 2. Chimerism and Tolerance: A Historical Perspective

The biologic basis for chimerism as a means to tolerance induction resulted from an observation in nature. In 1945 Owen demonstrated that a naturally occurring state of mixed chimerism occurred among freemartin fraternal bovine twins who shared a common placenta [20]. In 1953 Billingham and colleagues showed that these chimeric twins were later tolerant to skin grafts from their twin siblings while third-party grafts were readily rejected [21]. Many attempts at the induction of chimerism as a platform for tolerance induction have been undertaken in both small and large animal preclinical models and clinically. Lifelong tolerance of full thickness skin allografts across major histocompatibility barriers following nonmyeloablative conditioning and donor bone marrow with the induction of durable chimerism was demonstrated by Slavin and colleagues, who also showed that tolerance of organ allografts could be accomplished without the requirement of sustained chimerism [22, 23]. Sustained mixed lymphohematopoietic chimerism was demonstrated after “mixed marrow transplantation” involving the infusion of T-cell depleted autologous and allogeneic marrow following myeloablative conditioning in murine and miniature swine models which resulted in a tolerance of donor skin allografts [24, 25]. Reduced intensity transplant approaches in small and large (swine and primate) models using cyclophosphamide or low dose total body radiation and* in vivo* T-cell depletion showed that mixed chimerism was essential to the development of tolerance, but tolerance was achievable even if the chimerism was only transient [8, 10]. Evidence from multiple studies has shown that the allograft itself may be instrumental in the maintenance of donor specific unresponsiveness even in the absence of durable chimerism [23, 26]. Clinically, sustained mixed chimerism, particularly following transplants across major MHC barriers, has been very difficult to achieve with only anecdotal evidence that this occurs [27].

## 3. Combined Kidney and Bone Marrow Transplantation for Patients with Malignancy

Several hematopoietic cell transplant strategies have been developed in the clinic for the induction of mixed lymphohematopoietic chimerism as an immunologic platform for adoptive cellular therapy via donor lymphocyte infusions and for inducing tolerance for organ transplantation. Based on a murine model of Sykes and colleagues, in which mixed lymphohematopoietic chimerism was reliably achieved following nonmyeloablative conditioning therapy and MHC fully mismatched murine transplants for both tolerance induction and for optimizing a graft versus tumor effect [9, 28], we evaluated a similar model in clinical trials for patients with advanced hematologic malignancies [27, 29]. Similar to the murine model, conditioning therapy consisted of cyclophosphamide, peritransplant* in vivo* T-cell depletion (initially with equine antithymocyte globulin), and thymic irradiation (to deplete intrathymic T-cells which were not eliminated by ATG) on day −1. Mixed lymphohematopoietic chimerism was nearly uniformly achieved and in the majority of cases conversion to full donor chimerism hematopoiesis occurred either spontaneously or after delayed DLI. Dramatic antitumor responses in patients with advanced hematologic malignancies were often observed.

## 4. Combined HLA-Matched Donor Bone Marrow and Kidney Transplantation for Patients with Malignancy

**Figure 1:**
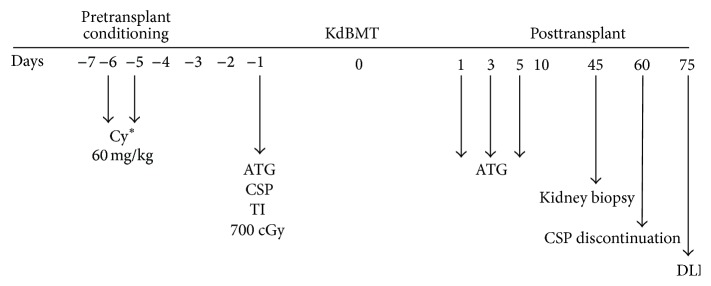
Combined HLA-matched bone marrow and kidney transplant. ^*∗*^Hemodialysis 14 hours after each Cy dose. Cy: cyclophosphamide; ATG: equine antithymocyte globulin; CSP: cyclosporine; cGy: centigray; DLI: donor lymphocyte infusion; KdMBT: combined kidney and bone marrow transplant.

Based on this experience and the results of the above described preclinical models of tolerance induction, we initiated a pilot trial of combined HLA bone marrow and kidney transplantation for patients with multiple myeloma and end stage renal disease [14, 16]. The initial schema for this trial is shown in Figure 1. Ten patients have undergone combined transplantation from HLA-matched related donors, with lead follow-up time of greater than 17 years. The feasibility and safety of this approach have been well established. There has been no transplant-related mortality. Six patients are alive, two of whom are currently in complete remission from 5+ to 13+ years after transplant. Of the other 4 patients two underwent a second hematopoietic cell transplant from their original donor for recurrent or progressive myeloma. One of the patients who received a second transplant is in a complete remission for more than 12 years after transplant. The other patient is being treated for recurrent central nervous system myeloma more than four years after the original combined transplant. One patient received a low dose total body irradiation and ATG preparative regimen after the original transplant was aborted due to sepsis and multiorgan failure. Two patients developed acute graft versus host disease (GVHD) and four developed chronic GVHD (two after a second allogeneic hematopoietic cell transplant). Five of ten patients had transient chimerism but, consistent with the preclinical primate model experience, sustained chimerism was not essential for durable tolerance. This experience demonstrated the proof of principle established in our preclinical models, namely, that combined bone marrow and kidney transplantation was feasible and that mixed or full donor chimerism, either transient or sustained, could result in long-term renal allograft acceptance without, in some cases, systemic immunosuppression. Because of cyclophosphamide related toxicities, including cardiotoxicity, and the observation that the one patient who received low dose TBI and ATG preparative therapy experienced minimal toxicity, the HLA-matched combined bone marrow and kidney transplant protocol (which now includes other hematologic malignancies and blood disorders) has been revised to substitute TBI 400 cGy for cyclophosphamide (NCT02158052). Given the limited number of patients with multiple myeloma with end stage renal disease who have willing and medically eligible sibling donors our experience has been expanded to include other hematologic malignancies and blood disorders for which allogeneic hematopoietic cell transplantation is indicated with end stage renal disease who have haploidentical related donors.

## 5. Combined Haploidentical Bone Marrow and Kidney Transplantation for Patients with Malignancy

With the advent of posttransplant high-dose cyclophosphamide based regimens, based on pioneering work at Hadassah University [30] and Johns Hopkins University [31], haploidentical HCT has become an accepted standard option for patients who lack fully matched related donors [31]. These modern regimens have managed to significantly decrease the significant rates of graft rejection, GVHD, and infection which complicated the historical experience with haploidentical HCT. The best described reduced intensity conditioning regimen for haploidentical HCT is comprised of fludarabine, low-dose cyclophosphamide, and 200 cGy of TBI followed by infusion of bone marrow or GCSF mobilized PBSCs on day 0. High-dose cyclophosphamide at 50 mg/kg/day is given on days +3 and +4 followed by tacrolimus and mycophenolate mofetil (MMF) starting on day +5 for prevention of GVHD. In a large multicenter cooperative group trial, 50 patients with various hematological malignancies were treated with such an approach. Impressively, no cases of severe (grades III-IV) acute GVHD, a 13% cumulative incidence at one year of chronic GVHD, and a 7% incidence of one year nonrelapse mortality were observed [32]. Using this regimen's framework, we are currently conducting an ongoing pilot clinical trial extrapolating the use of a posttransplant cyclophosphamide based regimen for combined hematopoietic stem cell and kidney transplantation from haploidentical related donors (NCT01758042).

**Figure 2:**
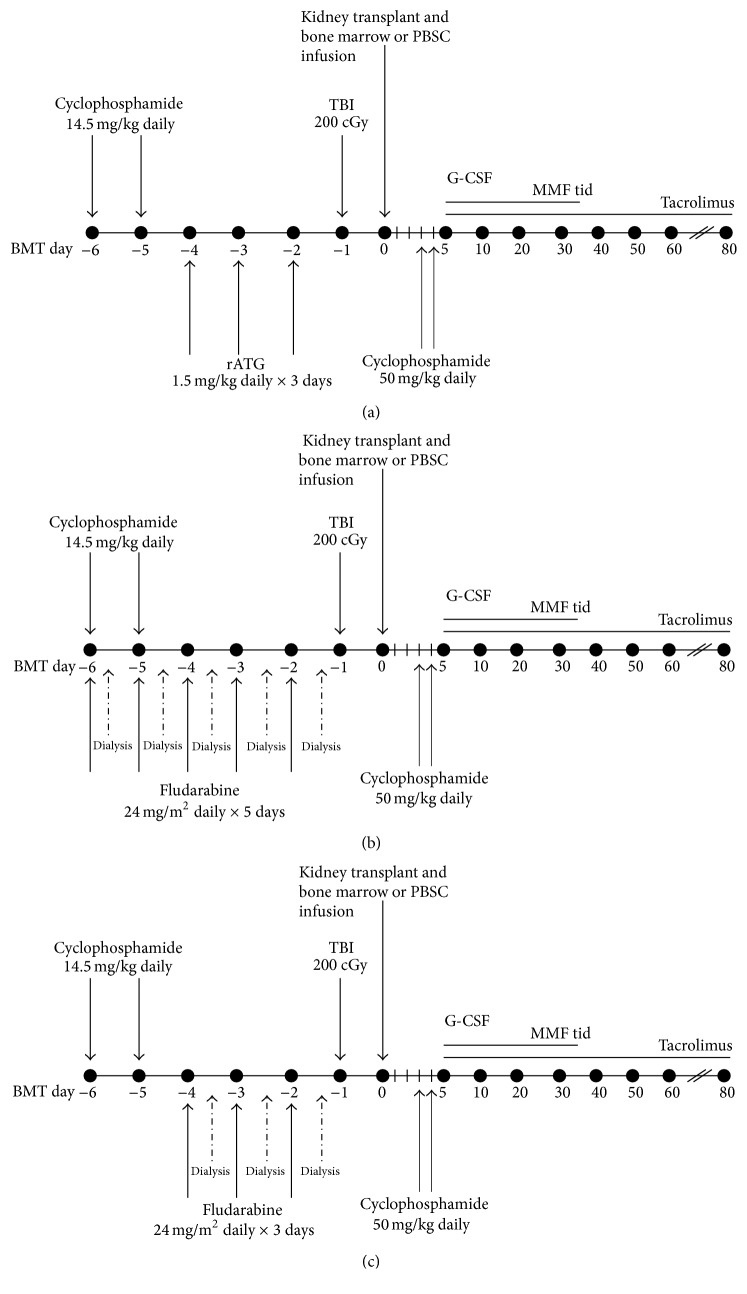
Schema for the 3 different versions (a, b, and c) of reduced intensity combined bone marrow and kidney transplantation using a haploidentical donor.

Given the significant renal clearance of fludarabine, we initially designed a reduced intensity conditioning regimen of rabbit ATG, low-dose cyclophosphamide, and 200 cGy TBI before simultaneous kidney and bone marrow transplant on day 0, which was then followed by standard posttransplant high-dose cyclophosphamide, tacrolimus, and MMF (Figure 2(a)). The first patient enrolled was a 67-year-old female with 17p deleted multiple myeloma and ESRD due to myeloma kidney. She tolerated therapy well with successful neutrophil engraftment on day +14, which was confirmed to be of predominant donor (>95%) origin, before secondary graft rejection occurred 10–14 days later. With supportive care, she ultimately recovered autologous hematopoiesis. She is now over 38 months after transplantation with no evidence of recurrent myeloma and normal renal function. She remains on low-dose tacrolimus. Lack of kidney allograft rejection despite hematopoietic graft rejection is similar to our observation among combined kidney and bone marrow transplant recipients without an underlying malignancy who received a different conditioning regimen (vide infra) and is consistent with the likelihood that the kidney may contribute to sustained tolerance.

Given the graft rejection experienced by our first patient, we revised the trial to eliminate ATG and add fludarabine (Figure 2(b)). While we had originally wished to avoid fludarabine in patients with significant renal dysfunction, we believed that a 20% dose reduction with daily hemodialysis would be safe and engender donor engraftment. This was based on several publications which studied the use of fludarabine in renal dysfunction [33, 34] including a recent combined bone marrow–kidney transplant trial delivering fludarabine safely in a similar manner [17].

The second patient was a 57-year-old female with multiple myeloma with ESRD from myeloma kidney. Her transplant course was relatively uncomplicated with successful donor engraftment and no evident fludarabine toxicities. She is now 24-months after transplantation with no evidence of recurrent myeloma, no chronic GVHD, and normal renal function and she was able to discontinue systemic immunosuppression approximately 8 months after transplantation. Our third patient was a 38-year-old male with relapsed non-Hodgkin lymphoma with longstanding ESRD of unclear etiology. His peritransplant course was complicated by significant nausea and vomiting during conditioning and poor renal recovery requiring a return to hemodialysis. At day +10, altered mental status was noted, and workup revealed epileptiform activity without any organized seizure activity. Cortical blindness developed around day +28 followed by bilateral lower extremity weakness and paraplegia. The entire constellation of symptoms and signs was attributed to fludarabine neurotoxicity. He continued with progressive neurological decline and poor hematopoietic recovery and expired 6 months after transplantation, ultimately from complications of fludarabine neurotoxicity.

Given the catastrophic toxicities and death of our third patient, we revised our protocol by (1) reduction of fludarabine to 24 mg/m^2^/day × 3 days (from 5 days), (2) lengthening of the hemodialysis sessions with a larger dialyzer, and (3) collection and analysis of fludarabine pharmacokinetics for each patient (Figure 2(c)). With this revised regimen, our fourth patient was a 52-year-old male with multiple myeloma and ESRD from myeloma kidney who underwent combined bone marrow and kidney transplantation from his haploidentical brother. His transplant course was fairly uncomplicated with full donor engraftment, normal renal function, and no GVHD. He is now more than 6 months after transplant and off immunosuppression. Fludarabine pharmacokinetics quantified by F-ara-A AUC as previously described [35] showed clearance of fludarabine comparable to that observed with full dose fludarabine given to patients with normal renal function (data not shown). Six more patients are scheduled to be enrolled and treated on this pilot study with this revised protocol.

As we have broadened the eligibility of our protocols to include patients with a variety of hematological malignancies and other life-threatening hematologic disorders using either HLA-matched or haploidentical related donors, inadvertent but inherent ethical issues have arisen. With conventional allogeneic HCT, 2-year rates of transplant-related mortality ranging from 5 to 20% depending on the regimen and donor are acceptable. In addition, there is generally no hesitation about performing an allogeneic HCT for patients with a high risk of disease relapse; indeed it is not unusual to quote long-term disease-free survival rates of 20–30% depending on the biology of the underlying disease. These risks are believed to be acceptable because (1) transplants are potentially curative and there are usually not comparable alternative treatment options and (2) donor hematopoietic stem cells are regarded as renewable with minimal long-term risk to the donor. With combined bone marrow and kidney transplantation, there is the added complexity of using a donor kidney, which is not a renewable organ and whose harvest accrues more risk for the donor. In addition, for advanced kidney disease, viable renal replacement therapy exists in the form of dialysis. Therefore, early mortality after kidney transplantation is not viewed as acceptable with rigorous review of each case to further mitigate such outcomes. If such an approach is to become more widely applicable, much discussion will certainly need to revolve around what is an acceptable risk and prognosis for these patients to qualify.

## 6. The Induction of Specific Tolerance for Patients without an Underlying Malignancy

The experience with combined bone marrow and kidney transplantations for patients with an underlying malignancy has shown the potential for specific tolerance induction and sustained antitumor responses. This strategy has involved the intentional induction of mixed chimerism with the eventual goal of full donor hematopoiesis either spontaneously or after DLI. In the context of a hematologic malignancy, GVHD is an acceptable complication, provided that it is not severe, owing to the theoretical accompanying graft versus tumor effect.

The broader application of tolerance induction strategies, however, is for patients with organ failure and without an underlying malignancy to avoid the deleterious effects of lifelong immunosuppressive therapy. Taking into account that GVHD is not an acceptable complication in this patient population and abundant experimental evidence has shown that mixed chimerism, even transiently, is capable of inducing sustained specific tolerance, strategies at the MGH have focused on inducing transient mixed chimerism as a platform for tolerance induction.

An early trial of nonmyeloablative allogeneic stem cell transplantation for refractory hematologic malignancies at the MGH involved preparative therapy with high-dose cyclophosphamide,* in vivo* T-cell depletion using an anti-CD2 humanized monoclonal antibody (MEDI-507), and thymic radiation and haploidentical bone marrow transplantation. In the first four patients lymphohematopoietic chimerism was only transient (range: 14 to 76 days) [36], and one of these patients achieved a dramatic and complete remission of her chemorefractory non-Hodgkin's lymphoma and is currently alive and disease-free more than ten years after the transplant [36, 37]. Transient chimerism was not the intent of the protocol, however, and the dosing and timing of MEDI507 were adjusted in subsequent trials to achieve a high level of sustained donor chimerism. The tolerability of the regimen in our first cohort of patients and the demonstration of uniform transient mixed chimerism were, however, considered to be ideal for a tolerance protocol.

Using this approach we initiated a combined haploidentical bone marrow and kidney transplant protocol for patients with renal failure and no underlying malignancy. Subsequent revisions of the protocol included the addition of rituximab to prevent humoral renal allograft rejection and short-course corticosteroids to ameliorate an “engraftment syndrome” (a cytokine “storm” occurring during the conversion of donor to host hematopoiesis and characterized by fever, fluid retention, and acute kidney injury) [38]. Of the first 10 patients treated with this approach, 7 achieved functional tolerance as defined by the withdrawal of systemic immunosuppression without renal allograft rejection for a minimum duration of 3 years following their transplant. Three patients subsequently (5–7 years after transplantation) required systemic immunosuppression, one for recurrent membranoproliferative glomerulonephritis and two for biopsy evidence of chronic humeral rejection. Four patients have remained off immunosuppression for >5 to >13 years after transplant [15, 18].

In an effort to avoid the toxicities of high-dose cyclophosphamide including severe gastrointestinal side effects and cardiotoxicity and to hopefully eliminate the “engraftment syndrome” that occurred in 9 of 10 patients treated on the previous protocol [15, 38], we substituted low-dose total body irradiation (150 × 2 cGy) for cyclophosphamide and continued rituximab and short-course posttransplant corticosteroids. Two patients have been treated on this protocol. Mixed chimerism in the first patient was of short duration. The “engraftment syndrome” did not occur. A late pancytopenia developed, however, but in both cases this was reversible. The first patient has been off systemic immunosuppression for 12 months with stable renal function, while the second who did not have donor hematopoietic chimerism developed subclinical humoral rejection and remains on immunosuppression.

Specific tolerance has been demonstrated* in vitro* by cellular assays of alloreactivity (mixed lymphocyte reaction (MLR) and cell mediated lympholysis (CML)) [15, 39, 40]. Early posttransplant patients showed global hyporesponsiveness but, by one year after transplant, showed return of third-party immunity with persistent anti-donor hyporesponsiveness. The mechanism of tolerance induction in these patients is uncertain. Central deletional (thymic) tolerance likely plays a minor role given the very transient nature of donor chimerism. An increase in circulating and intragraft CD4+ CD25+ FoxP3 expressing T-regulatory cells has been demonstrated suggesting a peripheral mechanism of tolerance induction [40]. Experimental evidence suggests that the kidney graft may also contribute to tolerance.

Other efforts at inducing specific tolerance have recently been reported. Leventhal and colleagues from Northwestern University have performed HLA-mismatched combined kidney and stem cell transplants from related and unrelated donors for renal failure of variable etiology [17]. This strategy has involved a cyclophosphamide, fludarabine, and low-dose TBI preparative regimen, the infusion of an GCSF mobilized peripheral blood stem cell product, which was engineered* ex vivo*, to remove GVHD producing T-cells and antigen presenting cells with retention of hematopoietic progenitors and facilitating cells (a population comprised principally of plasmacytoid precursor dendritic cells) on the day after the kidney transplant and then day 3 posttransplant high-dose cyclophosphamide to deplete alloreactive donor T-cells. Tacrolimus and mycophenolate mofetil for additional GVHD prophylaxis were administered. Despite the HLA disparity between donors and recipients, sustained hematopoietic engraftment has occurred in more than half of the patients resulting in the successful withdrawal of immunosuppressive therapy. GVHD has not been reported. Rapid return of CD4 and CD8 T central and effector memory cell populations has been observed and posttransplant vaccinations have been administered without loss of chimerism or allograft rejection [41].

The induction of tolerance has also been attempted at Stanford University by Scandling et al. for HLA-matched related and HLA-mismatched related and unrelated donor kidney and bone marrow transplantation [19]. Following kidney transplantation patients were treated with total lymphoid irradiation (TLI) and antithymocyte globulin and subsequent (day 11) infusion of a CD34 cell selected (with a variable number of T-cells) hematopoietic progenitor cell population. Variable donor chimerism, both in terms of percent donor cells and duration of chimerism, was achieved after HLA-matched donor transplantation. Seven of 16 patients had durable chimerism and were able to be successfully weaned from immunosuppressive drug therapy. In an initial cohort of 6 patients who received HLA-mismatched related or unrelated donor transplantation, none achieved durable chimerism or tolerance. In a subsequent cohort of patients receiving haploidentical donor transplants, higher CD34 cell and T-cell numbers were associated with higher levels of chimerism and their immunosuppression is currently being tapered. No GVHD has been observed in these patient cohorts.

## 7. Conclusions and Future Directions

Combined bone marrow and kidney transplants have demonstrated the feasibility and safety of this approach and proof of principle of tolerance induction after HLA-matched and mismatched transplantation. While the mechanisms of tolerance via persistent full donor chimerism are straightforward, those via transient chimerism are more complex which may involve both deletional and peripheral mechanisms [39, 40, 42]. Future studies will hopefully define further the mechanism of tolerance induction and define the optimal timing of hematopoietic cell and organ or tissue transplantation.

In order to further expand the application of tolerance approaches, several clinical trials have been proposed including widening the eligibility for combined transplants to patients with other hematologic disorders associated with renal failure such as sickle cell disease and extending tolerance approaches for other organs and tissues (such as limbs). In order to accomplish the latter, preparative therapy will have to be truncated to allow for either the immediacy of a cadaveric organ transplant or a delayed bone marrow transplant performed. While compression of the conditioning into one day resulted in unacceptable toxicity, promising results have been achieved with a tolerance approach in nonhuman primates. With this delayed tolerance approach, the recipient initially undergoes organ transplantation with conventional immunosuppression and then receives conditioning and bone marrow transplantation months later. In this model, long-term renal and lung allograft tolerance have been achieved [43, 44]. Based on these preclinical data, clinical trials using a delayed tolerance approach are planned.

## Figures and Tables

**Table 1:** 

Center	MGH	MGH	MGH	Stanford	Northwestern
Transplant type	HLA-matched related donor KdBMT for HM	Haploidentical donor KdBMT for HM	Haploidentical donor KdBMT for ESRD without malignancy	HLA-matched and haploidentical related and unrelated donor KdBMT for ESRD without malignancy	Haploidentical/mismatched related and unrelated donor KdBMT for ESRD without malignancy

Chimerism goal	FDC	FDC	Transient mixed	Sustained mixed	FDC

Results	Removal of IS in 5 of 10 patients (50%) Sustained antitumor responses in 30%	3 of 4 patients in remission, two likely tolerant	4 of 10 (40%) with sustained tolerance	Removal of IS in 44% (HLA-matched) 0% sustained tolerance (haploidentical or unrelated donor)	Removal of IS in 5 of 8 (63%) patients

KdBMT: kidney and bone marrow transplant; HM: hematologic malignancy; FDC: full donor chimerism; IS: immunosuppression.
